# Influence of the Location of Ascorbic Acid in Walnut Oil Spray-Dried Microparticles with Outer Layer on the Physical Characteristics and Oxidative Stability

**DOI:** 10.3390/antiox9121272

**Published:** 2020-12-14

**Authors:** Denisse Cáceres, Begoña Giménez, Gloria Márquez-Ruiz, Francisca Holgado, Cristina Vergara, Patricio Romero-Hasler, Eduardo Soto-Bustamante, Paz Robert

**Affiliations:** 1 Departamento de Ciencia de los Alimentos y Tecnología Química, Facultad de Ciencias Químicas y Farmacéuticas, Universidad de Chile, Santos Dumont 964, Independencia, Santiago 8380494, Chile; denicacer@ug.uchile.cl; 2 Departamento de Ciencia y Tecnología de los Alimentos, Facultad Tecnológica, Universidad de Santiago de Chile, Av. Ecuador 3769, Estación Central, Santiago 9170124, Chile; bego.gimenez@usach.cl; 3 Instituto de Ciencia y Tecnología de Alimentos y Nutrición (ICTAN-CSIC), José Antonio Nováis 10, 28040 Madrid, Spain; gmarquez@ictan.csic.es (G.M.-R.); f.holgado@ictan.csic.es (F.H.); 4 Instituto de Investigaciones Agropecuarias, INIA La Platina, Santiago Chile, Av. Santa Rosa 11610, La Pintana, Santiago 8831314, Chile; cristinavergarah@gmail.com; 5 Departamento de Química Orgánica y Fisicoquímica, Facultad de Ciencias Químicas y Farmacéuticas, Universidad de Chile, Santos Dumont 964, Independencia, Santiago 8380494, Chile; xumi@ug.uchile.cl (P.R.-H.); esoto@ciq.uchile.cl (E.S.-B.)

**Keywords:** walnut oil, microencapsulation, three-fluid nozzle, oxygen scavenger, spray drying, sodium alginate

## Abstract

Purified walnut oil (PWO) microparticles with Capsul^®^ (C, encapsulating agent), sodium alginate (SA) as outer layer and ascorbic acid (AA) as oxygen scavenger were obtained by spray drying using a three-fluid nozzle. AA was incorporated in the inner infeed (PWO-C(AA)/SA), in the outer infeed (PWO-C/SA(AA)) and in both infeed (PWO-C(AA)/SA(AA)). PWO-C(AA)/SA (4.56 h) and POW-C(AA)/SA(AA) (2.60 h) microparticles showed higher induction period than POW-C/SA(AA) (1.17 h), and lower formation of triacylglycerol dimers and polymers during storage (40 °C). Therefore, AA located in the inner infeed improved the oxidative stability of encapsulated PWO by removing the residual oxygen. AA in the SA outer layer did not improve the oxidative stability of encapsulated PWO since oxygen diffusion through the microparticles was limited and/or AA weakened the SA layer structure. The specific-location of AA (inner infeed) is a strategy to obtain stable spray-dried polyunsaturated oil-based microparticles for the design of foods enriched with omega-3 fatty acids.

## 1. Introduction

Walnut oil (WO) is gaining interest in food and pharmaceutical industries, due to its high content in α-linolenic acid (ALA, 10–13%) and other bioactive compounds (phytosterols, polyphenols and tocopherols) [1,2]. Several beneficial health effects have been associated with ALA, such as cardioprotective, anticancer, neuroprotective, anti-osteoporotic and anti-inflammatory properties [3]. The daily intake of ALA recommended by several organizations such as International Society for the Study of Fatty Acids and Lipids (ISSFAL) is 0.5–0.7% of total energy [4]. However, the ALA consumption by the general population is low and, therefore, the food industry is interested in the incorporation of healthy ingredients based on ALA-rich vegetable oils into food products [1,2], and particularly in encapsulated oils, given that oils have poor water solubility and high susceptibility to oxidative deterioration.

Microencapsulation technology allows protection of polyunsaturated oils, highly susceptible to oxidative deterioration, from environmental (oxygen, light, humidity and temperature), food (pH, enzymes) and gastrointestinal conditions (pH, enzymes), providing an effective barrier against deteriorating factors [5]. Even though encapsulation of WO is still scarce, there are references on microencapsulation by freeze-drying [6] and spray-drying [3,7,8]. A variety of biopolymers have been reported for the encapsulation of ALA-rich oils by spray-drying, the most common method for encapsulation of marine and vegetable oils, but only a few of them have been used for WO: maltodextrin with hydroxypropyl methylcellulose [8], a mixture of gum arabic, maltodextrin and gelatin [7], skim milk powder (SMP), and SMP with maltodextrin [3].

Although microencapsulation protects the oils from degradation, microencapsulated oils have shown higher degradation than non encapsulated oils in some studies [8]. This may be due to the oxidation of the encapsulated oil promoted by the oxygen incorporated in the emulsion during the homogenization process and its diffusion within the microparticles [9]. The addition of antioxidants or antioxidant extracts to the infeed to avoid the oxidation of encapsulated oils has been tested, but this strategy has been little studied [8,10]. Furthermore, microencapsulation offers an opportunity to design site-specific delivery WO microparticles, where the encapsulating agent plays an important role, maintaining the stability and functionality of encapsulated oils, and allowing their delivery at the absorption site. Sodium alginate (SA) is a linear polysaccharide composed of alternating blocks of 1–4 linked α-L-guluronic and β-D-mannuronic acid residues, capable of forming a gel structure. SA is an interesting encapsulant agent for oil encapsulation, because it is stable at acidic pH but dissolves under mild alkaline conditions. Thus, the structure of SA remains intact in the stomach (acidic pH) but it is swollen and/or disintegrated in the small intestine (alkaline pH), releasing the fatty acids [11]. Thus, SA can be used as a polymer with intestinal-specific delivering, but its high viscosity at low solid content (2%) limits its use as encapsulating agent in spray-drying. In this context, the use of three-fluid nozzles in spray-drying may be a suitable strategy to obtain WO microparticles with SA as external-layer. In the three-fluid nozzle, two feed solutions (inner and outer feed solutions) can be individually pumped through two separate concentric channels, whereas air flows through the third channel. The solution sprayed through the outer channel covers the solution sprayed through the inner one when feed solutions meet at the tip of the nozzle during the atomization process, generating a microparticle where two layers can be completely differentiated [12,13]. A novel strategy not yet studied in spray-dried microparticles, consisting of using oxygen scavenger agents to remove oxygen from powder microparticles, was addressed in this work. Ascorbic acid (AA) is a natural oxygen scavenger, able to remove the residual oxygen [14]. Besides, it is widely used as food ingredient with biological activity of vitamin C and generally recognized as safe (GRAS).

In this study, WO microparticles with an internal core composed of a WO-in-water emulsion, and an outer layer composed of SA were designed using a three-fluid nozzle. The objective of this work was to determine the physical characteristics of the WO microparticles designed and to study the effect of the location of ascorbic acid (inner infeed, outer infeed and in both infeed) as oxygen scavenger on the lipid oxidation of microencapsulated WO.

## 2. Materials and Methods

### 2.1. Materials

Walnut oil was purchased from Nutra Andes Ltd.a. (Valparaíso, Chile). The major fatty acids were 5.6 ± 0.9% (C16:0), 2.1 ± 0.2% (C18:0), 16.8 ± 2.0% (C18:1 n-9), 59.7 ± 1.9% (C18:2 n-6), 14.78 ± 0.4% (C18:3 n-3). The initial values of free acidity and peroxide value were 0.06 ± 0.0% oleic acid and 1.4 ± 0.1 mEq O_2_/kg oil, respectively. Soy lecithin (SL) (Epikuron 145V) was supplied by Blumos Ltd.a. (Santiago, Chile), aluminum oxide 90 active acidic (0.063–0.200 mm) was obtained from Sigma-Aldrich (Santiago, Chile), Capsul^®^ (C) was obtained from Mathiesen S.A. (Santiago, Chile), sodium alginate (SA) was obtained from Alginatos Chile S.A. (Paine, Chile), L-ascorbic acid (AA, 99%) and solvents of analytical grade (ethanol, acetone, hexane, chloroform, and sulfuric acid) were purchased from Merck (Santiago, Chile).

### 2.2. Methods

#### 2.2.1. Walnut Oil Purification

Walnut oil was antioxidant-stripped by open column adsorption chromatography (25 cm × 2 cm) packed with aluminum oxide according to [15]. Previously, aluminum oxide was activated in an oven at 180 °C for 3 h. The purified walnut oil (PWO) was analyzed by HPLC to confirm the absence of tocopherols [16,17]. 

#### 2.2.2. Purified Walnut Oil (PWO) Characterization 

The peroxide value [18] and free acidity [19] were evaluated according to AOCS. The induction period (IP) is the time up to which secondary lipid oxidation products arise and characterizes the oxidative stability of oils and fats. This parameter was determined using a Rancimat 892 equipment (Metrohm Ltd., Herisau, Switzerland) at 100 °C and an air flow of 20 L/h.

#### 2.2.3. PWO Emulsion Preparation

Statistical design. PWO-in-water emulsion was performed according to a Box-Behnken experimental design (15 runs, 12 experimental points and three central points). SL content (2–6% of PWO), homogenization time (HT, 1–5 min) and homogenization speed (HS, 11,000–20,000 rpm) were the independent variables. Droplet size (D_[4,3]_), and lipid oxidation evaluated as thiobarbituric acid reactive substances (TBARs) were the dependent variables.

Experimental data were fit to a second-order regression model. All the experiments were conducted randomly to avoid systematic bias. Response surface methodology (RSM) was applied at each independent variable and the multiple response optimization was performed using the desirability function, where droplet size and TBARs values were minimized.

Preparation and characterization of PWO emulsion. PWO-in-water emulsions (6 g) were prepared by dispersing SL (0.05–0.16 g) in distilled water (3.17–3.28 g) at 40 °C by stirring at 500 rpm for 20 min. The dispersion was added to PWO (2.67 g) and the mixture was homogenized with a Polytron PT-2100 (Kinematica AG, Luzern, Switzerland). Droplet size of PWO emulsions was determined by laser diffraction, using a particle size analyzer (Mastersizer X, Malvern Instruments, Malvern, UK), and expressed as D_[4,3]_. Lipid oxidation was evaluated by TBARs, according to [20].

#### 2.2.4. Encapsulation of PWO Emulsion by Spray-Drying

Statistical design. Encapsulation of PWO-in-water emulsion with C (PWO-C) was performed by spray drying according to a central composite design plus star point (12 runs, 8 experimental points and four central points). The independent variables were the inlet air temperature (120–180 °C) and the PWO:C ratio (1:1–1:5). The dependent variables were the encapsulation efficiency (EE) of PWO, the yield (Y) and the induction period (IP). RSM was applied for each independent variable. Experimental data were fit to second-order regression model. Optimization was performed using the desirability function, where all the response variables were maximized.

Preparation and characterization of PWO microparticles. The infeed emulsion (100 g) was prepared as follows: the PWO-in-water emulsion obtained under optimal conditions (13.87 g) was immediately incorporated into a solution of C (2.32–21.68 g) in distilled water (64.45–83.85 g). The resulting dispersion was fed into a mini-spray-dryer (B-290, Büchi, Flawil, Switzerland). The drying conditions were: airflow of 600 L/h, rate of feeding of 2 mL/min, atomization pressure of 20 psi, and inlet air temperature in the range from 120 °C to 180 °C. PWO-C microparticles obtained were stored at −20 °C until analysis.

Encapsulation efficiency (EE): The surface oil was determined according to Shamaei et al. [3]. Briefly, hexane (10 mL) was added to powder microparticles (1 g) and filtered through a filter paper (Whatman No 1). The filter was rinsed three times with 10 mL of hexane. The solvent was removed with a rotatory evaporator (R250, Büchi). The encapsulation efficiency (EE) of PWO was calculated according to Equation (1):

(1) $$\mathrm{EE}\;(\%)=\frac{\mathrm{Total}\;\mathrm{oil}\;-\mathrm{Surface}\;\mathrm{oil}}{\mathrm{Total}\;\mathrm{oil}}\times 100$$

where total oil corresponds to theoretical total oil in the microparticles.

Induction period (IP): IP was determined by Rancimat as described in Section 2.2.2.

Yield (Y) was determined according to Equation (2):

(2) $$Y\;(\%)=\frac{\mathrm{powder}\;\mathrm{obtained}\;\mathrm{after}\;\mathrm{spray}\;\mathrm{drying}\;\left(g\right)}{\mathrm{solid}\;\mathrm{content}\;\mathrm{in}\;\mathrm{infeed}\;\mathrm{spray}\;\mathrm{drying}\;\left(g\right)}\;\times 100$$

#### 2.2.5. Formation and Confirmation of a Sodium Alginate Outer Layer Using a Three-Fluid Nozzle.

The inner infeed (PWO-C, 35 g) was prepared under optimal conditions: PWO (1.4 g), SL (0.06 g), C (7.54 g) and water (26 g). The outer infeed (350 g) consisted of a SA solution (7g SA). Both inner and outer infeed were fed into a Büchi B-290 spray dryer (Flawil, Switzerland) using a three-fluid nozzle with an inner:outer infeed ratio of 1:10. The drying conditions were: airflow of 600 L/h, atomization pressure of 20 psi, rate of feeding of 0.12 mL/min and 1.2 mL/min for inner infeed and outer infeed, respectively, and inlet air temperature of 114 °C. Thus, PWO-C microparticles with a SA outer layer (PWO-C/SA) were obtained.

##### Confirmation of Sodium Alginate Outer-Layer Using a Three-Fluid Nozzle

The formation of the SA outer layer was confirmed by confocal laser scanning microscopy (CLSM; LSM 700, Carl Zeiss, Ulm, Germany), using the software ZEN 2012 (Blue Edition, Carl Zeiss, Ulm, Germany). 5(6)-carboxyfluorescein (0.5 mL, 25 mM) was incorporated in the outer infeed of SA. The microparticles were placed onto a microscope slide, covered with a cover glass and subjected to the excitation wavelength of 5(6)-carboxyfluorescein (490 nm), while the fluorescent signal was collected at 520 nm.

#### 2.2.6. Incorporation of Ascorbic Acid (AA) in PWO-C/SA Microparticles

Ascorbic acid (10, 20, 30, 40 and 50 mM) was added to three specific locations of the PWO-C/SA system (Figure S1): in the inner infeed (PWO-C(AA)/SA), in the outer infeed (PWO-C/SA(AA)), and both in the inner and the outer infeed (PWO-C(AA)/SA(AA)).

#### 2.2.7. Characterization of PWO-C/SA Microparticles with Ascorbic Acid

Microparticles with AA ((PWO-C(AA)/SA, PWO-C/SA(AA) and PWO-C(AA)/SA(AA)) and without AA (PWO-C and PWO-C/SA) were characterized according to the following analyses.

##### Water Activity, Moisture Content and Hygroscopicity

The moisture content was determined using an infrared moisture analyzer (PMC50, Radwag, Miami, FL, USA). Water activity (a_w_) was measured by the determination of the dew point at 20 ± 0.3 °C (Hygrolab 2, Rotronic, Hauppauge, NY, USA). The hygroscopicity was measured by the desiccator method, using sodium sulphate under controlled relative humidity [21].

##### Ascorbic Acid Recovery

The content of AA in PWO-C/SA microparticles was determined by HPLC [22], composed of a Merck Hitachi L6200 pump, a photodiode-array detector (996 Waters, Milford, MA, USA) and a C18 column (5 µm, 4.6 mm i.d. × 250 mm, Symmetry, Waters, Dublin, Ireland). Sulphuric acid 0.01% (*v*/*v*) was used as mobile phase at a flow of 0.8 mL/min. AA was detected at 254 nm and quantified using a calibration curve (0.49–4.9 mM, R^2^ = 0.99).

##### Morphology of the Microparticles

The microparticle morphology was evaluated using a scanning electron microscopy (SEM). Samples were covered with gold/palladium using a PS 10E vacuum evaporator (Varian, Vineland, NJ, USA) and analyzed using a LEO 1420VP microscope (LEO Electron Microscopy Ltd., Cambridge, UK) operated at 20 kV. The software used for the SEM imaging was EDS 7424 (Oxford Instruments, Oxford, UK).

##### Induction Period (IP)

The IP of the microparticles (2 g) was determined using a Rancimat 892 instrument (Metrohm Ltd., Herisau, Switzerland) as described in Section 2.2.2.

##### Thermal Analysis

The thermal behavior was characterized by modulated differential scanning calorimetry (MDSC), using a Q20 differential scanning calorimeter (TA Instruments, New Castle, DE, USA). The samples were placed in sealed aluminum pans and measured by heating from −40 to 200 °C, at a rate of 1.5 °C/min and with a modulation regime of 1.5 °C every 90 s. The glass transition temperature (Tg) and melting points were obtained with the Universal analysis 2000 software (v4.5A, TA Instruments, New Castle, DE, USA).

#### 2.2.8. Oxidative Stability Assays

Microparticles (2.5 g) with AA in different location (PWO-C/SA(AA), PWO-C(AA)/SA and PWO-C(AA)/SA(AA)) and without AA (PWO-C/SA) were placed in open Petri dishes (10 cm diameter) and stored in the dark at 40 ± 1 °C in a forced–air oven (BE 500, Memmert, Schwabach, Germany). Petri dishes (in triplicate) were removed every 6 h. In each sample, the encapsulated PWO was extracted to determine triacylglycerol dimers and polymers.

##### Encapsulated Oil Extraction

Firstly, the surface oil was removed from the microparticles (2.5 g), following the same procedure described in Section 2.2.4. Then, the encapsulated oil within the microparticles was extracted by disrupting the microparticles in a mortar with water (0.5 mL), until a clotted mass was obtained. Then, diethyl ether (20 mL) was added to the clotted mass and the mixture was centrifuged. The extraction was repeated three times, the supernatants were joined, and the solvent was removed using a nitrogen stream at 40 °C until constant weight.

##### Determination of Triacylglycerol Dimers and Polymers

Oil extracted (50 mg) from microparticles was dispersed in hexane (2 mL), and directly analyzed by HPSEC, using a chromatograph equipped with a Waters 510 pump (Waters, Milford, MA, USA), a refractive index detector (HP1037A, Agilent Technologies, Santa Clara, CA, USA), and a Rheodyne 7725i injector (10 µL sample loop). Two columns (100 Å and 500 Å; 5 µm, 0.77 cm i.d × 25 cm, Agilent Technologies, Santa Clara, CA, USA), packed with porous highly cross-linked styrene-divinylbenzene copolymers were connected in series. Tetrahydrofuran was used as mobile phase at a flow of 1 mL/min. Triacylglycerol polymers and triacylglycerol dimers were quantified as peak area percentage [23].

#### 2.2.9. Statistical Analysis

All the experiments were performed by triplicate. The results were reported as average ± standard deviation (SD) from three replicates. One-way ANOVA was applied to determine the statistical differences among samples, using the Statgraphics software (version 7.0; Statistical Graphics Corporation, Warrenton, VA, USA).

## 3. Results and Discussion

γ-Tocopherol was the main tocopherol in WO (355 ± 45 mg/kg oil), whereas the content of α-tocopherol and δ-tocopherol (11 ± 1 mg/kg oil and 25 ± 1.5 mg/kg oil, respectively) was noticeably lower. Tocopherols from WO were removed by purification. Both peroxide value and free acidity significantly (*p* < 0.05) decreased with WO purification process, from 1.91 ± 0.01 mEq O_2_/kg oil and 1.43 ± 0.10% in WO to 0.06 ± 0.01 mEq O_2_/kg oil and 0.25 ± 0.01% in PWO. Furthermore, PWO showed lower IP values (0.41 ± 0.01 h) than WO (5.58 ± 0.12 h), because of the removal of tocopherols.

### 3.1. PWO Emulsion Preparation

The encapsulation of PWO by spray-drying requires the previous preparation of a PWO-in-water emulsion. The Box-Behnken experimental design and the ANOVA for the preparation of the PWO-in-water emulsion are shown in Supplementary Material (Table S1). Droplet size (D_[4,3]_) and TBARs values were in the range 10.5–63.7 µm and 0.401–0.745 mg MDA/kg oil, respectively. The ANOVA for droplet size showed that the linear form of SL content, HT and HS, as well as the quadratic form of HT and the interaction between SL content and HT were significant (*p* < 0.05). The response surface graphics for droplet size (Figure S2a–c) showed that the SL content was the most important factor in determining the droplet emulsion size, and the higher the SL content the smaller the droplet size. However, the higher the HT, the higher the droplet size, especially at low HS and low SL content. HS did not have a noticeably effect on droplet size in the range studied. Commonly, small droplet emulsion size is desirable since it has been related with higher emulsion stability [3].

In the case of TBARs, only the quadratic forms of SL content, HT and HS were significant (*p* < 0.05). The response surface graphics (Figure S2d–f) showed that the lowest lipid oxidation measured by TBARs was found at intermediate values of the SL content and at the lowest and highest values of HS. However, HT did not have a noticeable effect on TBARs values in the studied range. High homogenization speed involves high shear stress during the emulsification process that has been associated with higher incorporation of oxygen in the oil-in-water emulsion, which promotes oxidation of polyunsaturated lipids [24,25], increasing TBARs values. The model explained 89.6% and 76.6% of the variability (R^2^ adjusted for degrees of freedom) in droplet size (D_[4,3]_) and TBARs, respectively, with residual values below 6.0 and non-significant (*p* > 0.05) lack of fit.

**Figure 1:**
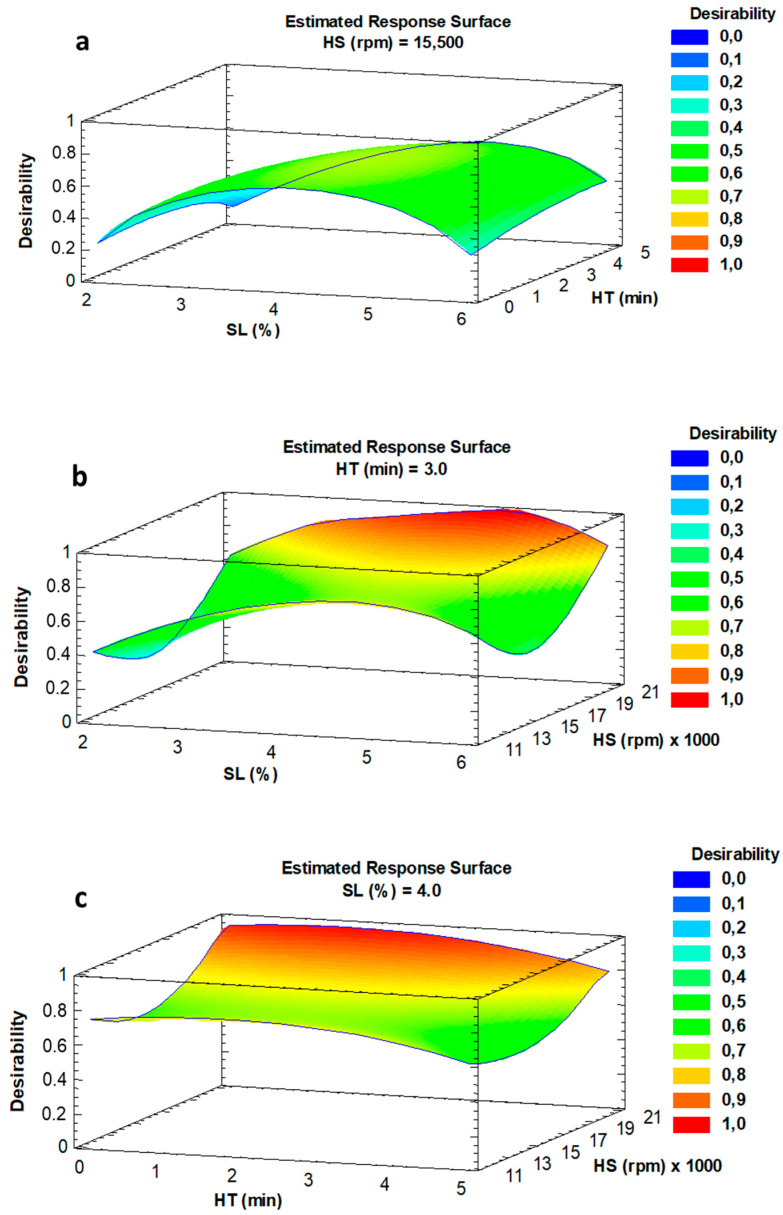
Desirability function for PWO emulsion preparation (**a**–**c**). SL: soy lecithin; HT: homogenization time; HS: homogenization speed

The desirability function was used for the multiple response optimization, minimizing both droplet size and TBARs response variables (Figure 1a–c). The highest value for desirability (0.93) was achieved with 0.12 g SL (4.4% of PWO), HT of 2 min and HS of 20,000 rpm, values within the experimental domain studied. The optimal conditions obtained in this study were within the range reported for the SL content (2.5–15% of oil; [8,26,27], HT (1–15 min) and HS (7200–24,000 rpm) [7,28,29] used in the preparation of oil-in water emulsions with PUFA rich oils that are further spray dried. The values predicted by the model for D_[4,3]_ and TBARs under these optimal conditions were 12.60 µm and 0.43 mg MDA/kg oil, respectively.

### 3.2. PWO Encapsulation by Spray Drying

The central composite plus star design and the ANOVA for the encapsulation of PWO-in-water emulsion with C by spray drying are shown in Supplementary Material (Table S2). The EE values of PWO ranged between 72.4% and 90.9%, IP values between 0.22 h and 1.44 h, and Y values between 16.1% and 38.5%.

**Figure 2:**
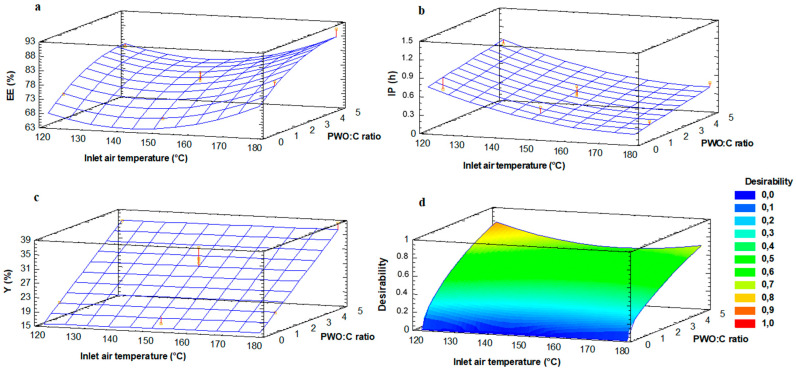
Response surface graphics for encapsulation efficiency (EE; (**a**)), induction period (IP; (**b**)), yield (Y; (**c**)) and desirability function (**d**) for PWO encapsulation by spray drying.

The inlet air temperature, both in its linear and quadratic form, and the linear form of PWO:C ratio were significant (*p* < 0.05) in EE and IP, whereas the quadratic form of PWO:C ratio was only significant (*p* < 0.05) for EE. In the case of Y, only the linear form of PWO:C ratio was significant (*p* < 0.05). As it is shown in the response surface graphic (Figure 2a–c), the higher the content of C the higher the values of EE, both at high and low temperatures (Figure 2a). A higher solid content in the infeed and/or inlet air temperature have been associated with higher EE of the oil-in-water emulsion and lower amount of unencapsulated oil at the surface of the powder microparticles, which is highly prone to oxidation [30]. Y values increased with increasing contents of C, independently of the inlet air temperature used (Figure 2c), suggesting that the outlet temperature is more important on Y, since powders with a higher moisture content are more easily retained in the drying chamber [31]. Furthermore, the lower the temperature the higher the IP values, since low drying temperatures improve the oxidative stability of oil (Figure 2b). The model explained 89.3%, 80.0% and 84.7% of the variability (R^2^ adjusted for degrees of freedom) in EE, IP and Y, respectively. The residual values (difference between the calculated and experimental results) were below 6.0 and the lack-of-fit was not significant (*p* > 0.05) in any case, indicating that the model fit well to the experimental data.

The desirability function was applied for the multiple response optimization, maximizing all the response variables (EE, IP and Y). Figure 2d shows the desirability graph for the encapsulation of PWO-in-water emulsion. The highest value for desirability (0.86) was reached with an air inlet temperature of 114 °C and a PWO:C ratio of 1:5.42, both within the domain studied but close to the star point. These drying conditions are within the range used for the encapsulation of PUFA rich oils in the literature, where values between 110 °C and 220 °C and ratios between 1:1 and 1:10 have been reported as air inlet temperature and oil:encapsulating agent ratio, respectively [5,7]. The values predicted by the model for EE, IP and Y under these optimal conditions were 84.3%, 1.15 h and 34.3%, respectively.

### 3.3. Study of the SA Outer Layer Formation in PWO-C Microparticles

**Figure 3:**
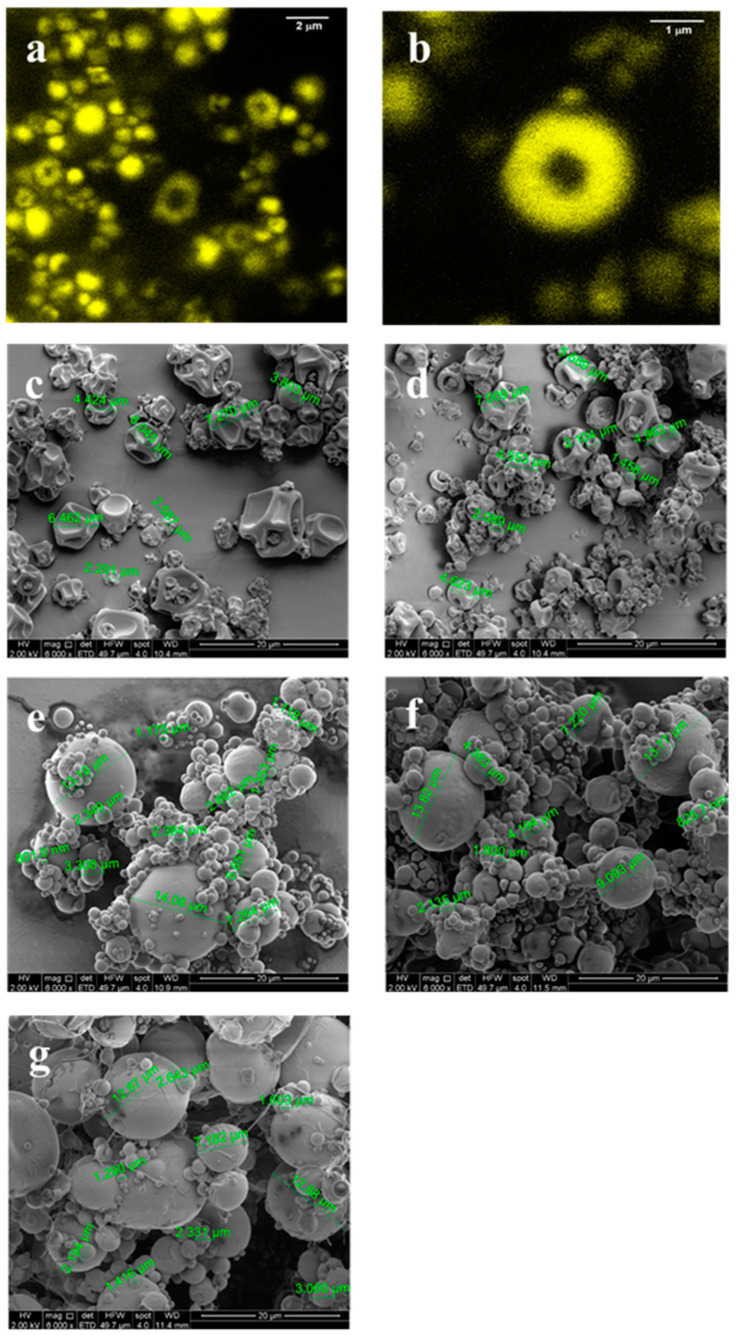
CLSM micrographs for PWO-C/SA (**a**,**b**). SEM micrographs of PWO-C microparticles (**c**), PWO-C/SA microparticles (**d**), PWO-C(AA)/SA microparticles (**e**), PWO-C/SA(AA) microparticles (**f**) and PWO-C (AA)/SA(AA) microparticles (**g**).

PWO-C microparticles with a SA outer layer (PWO-C/SA) were obtained using a three-fluid nozzle. Microphotographs of PWO-C/SA microparticles labelled with carboxyfluorescein are shown in Figure 3a,b). A yellow ring can be clearly seen around the PWO-C microparticles, confirming the formation of the outer layer of SA.

### 3.4. Effect of the Concentration and Localization of AA on the Oxidative Stability of Encapsulated PWO

**Figure 4:**
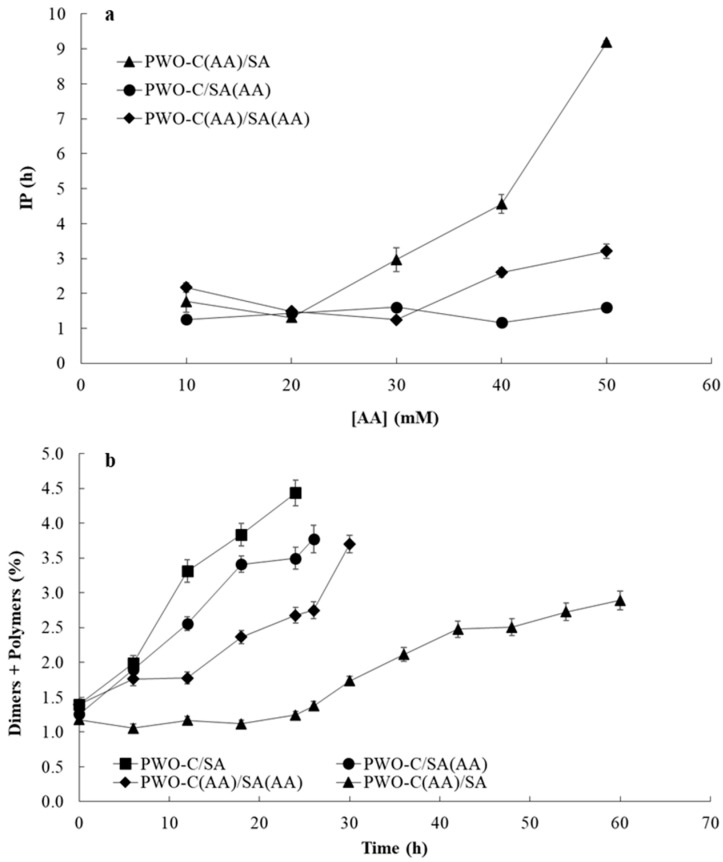
(**a**): Evolution of IP with increasing concentrations of AA in the three locations of PWO microparticle system: inner infeed (PWO-C(AA)/SA), outer infeed (PWO-C/SA(AA)), in both infeed (PWO-C(AA)/SA(AA)). (**b**): Formation of triacylglycerol dimers and polymers during storage of microparticles at 40 ± 1 °C in PWO-C/SA, PWO-C(AA)/SA, PWO-C/SA(AA) and PWO-C(AA)/SA(AA).

Oxygen promotes the deterioration not only of bulk lipids but also encapsulated lipids by complex oxidative reactions. Thus, a variety of oxygen scavengers and/or absorbents have been used in food preservation [14]. AA, which was used in this study as oxygen scavenger to remove the residual oxygen and to avoid the oxygen diffusion within the microparticles, was incorporated in three specific locations in the PWO-C/SA system: in the inner infeed (PWO-C(AA)/SA), in the outer infeed (PWO-C/SA(AA)), and in both infeed (PWO-C(AA)/SA(AA)). Figure 4a shows the evolution of IP with increasing concentrations of AA in the three locations studied. IP values were similar (around 1–2 h, *p* > 0.05) in the three systems until 20 mM of AA, but significant differences (*p* < 0.05) were found among the microparticle systems from 30 mM to 50 mM of AA. In the case of PWO-C(AA)/SA, AA was incorporated in the inner infeed, where AA was able to remove the residual oxygen that was incorporated during the emulsification process, showing the highest IP values from 30 mM. In contrast, the incorporation of AA in the SA outer infeed (PWO-C/SA(AA) microparticles) did not increase IP values in the range of the AA concentrations studied, suggesting that the AA in the SA outer layer did not have any significant effect on oxidative stability of the microparticles. This result may be explained because microparticles were analyzed immediately after their preparation, where the diffusion of oxygen from surrounding air into the SA outer layer in the microparticles was negligible. However, the incorporation of AA in both inner and outer infeed (PWO-C(AA)/SA(AA), led to a significant (*p* < 0.05) increase of IP from 40 mM, attributed only to the effect of AA incorporated in the inner infeed.

Therefore, the specific location of AA influenced the oxidative stability of the encapsulated PWO, measured by IP, where the inner infeed was the most effective location. From these results, 40 mM of AA was selected (lower AA content with differences among systems) to be incorporated in the three specific locations of the PWO-C/SA microparticles obtained under optimal conditions.

### 3.5. Characterization of PWO Microparticles Obtained under Optimal Conditions

#### 3.5.1. Encapsulation Efficiency (EE)

The EE represents the retention of the PWO-in-water emulsion within the microparticles, forming a matrix structure. EE is an important parameter in oil encapsulation by spray drying, which usually is related to the increase of oil oxidative stability by reducing the amount of surface oil that is exposed to environmental conditions [5]. The formation of a SA outer layer led to significant (*p* < 0.05) higher EE values of PWO, as shown in Table 1, where PWO-C/SA, PWO-C(AA)/SA, PWO-C/SA(AA) and PWO-C(AA)/SA(AA) systems showed EE values of PWO over 90%. Moreover, the incorporation of AA in specific locations of the PWO-C/SA microparticles did not affect the EE of PWO (*p* > 0.05). The formation of an outer layer surrounding PWO microparticles helped encapsulating the PWO that remained on the surface. Similar values of EE have been reported for encapsulation of WO using SMP and SMP + Tween-80 (90% and 91%, respectively [3]). However, lower EE values have been reported when WO was encapsulated with maltodextrin and hydroxypropyl methylcellulose (73% [8]), a mixture of Arabic gum, maltodextrin and gelatin (14.7–44.1% [7]), or a mixture of SMP and maltodextrin (56.2% [3]).

#### 3.5.2. Induction Period (IP)

PWO-C microparticles showed higher IP values (0.99 h, Table 1) than PWO (0.41 h). A protective effect of encapsulation of oils by spray drying on lipid oxidation has been reported in several studies, since the encapsulating agent may act as a physical barrier to prevent degradation triggered by moisture, metal ions, oxygen, and heat [1,2]. The formation of a SA outer layer increased IP values of all the PWO microparticles, indicating a protective effect of the SA coating, except in the microparticles where AA was only incorporated in the SA layer (PWO-C/SA(AA)). This result could be explained because some reducing compounds, such as AA, induce alginate degradation through the formation free radicals that cause the breakdown of alginate chains [32]. Therefore, the lowest oxidative stability of PWO-C/SA(AA) among the systems with SA outer layer could be due to a pro-oxidant effect of AA, in this case concentrated in the SA layer, resulting in a reduction of the mechanical resistance of the SA outer layer, and hence in the protective effect on PWO oxidation. Furthermore, the absence of oxygen diffusion through the microparticles may be also related with the lower IP values found in PWO-C/SA(AA) microparticles, as it was discussed in Section 3.2. The systems with AA incorporated in the infeed emulsion, PWO-C(AA)/SA and PWO-C(AA)/SA(AA), showed higher IP values, 4.56 h and 2.6 h, respectively, because of the oxygen scavenging effect of AA in the infeed emulsion. IP values of PWO-C(AA)/SA were about twice than those found in PWO-C(AA)/SA(AA), since all the AA (40 mM) was incorporated in the infeed emulsion in the first case, while 20 mM of AA was incorporated both in the infeed emulsion and in the SA outer layer in the second case. Therefore, the effect on IP value in the PWO-C(AA)/SA(AA) microparticle system may be only attributed to the action of AA in the inner infeed. Some natural antioxidant extracts, such as rosemary and murta leaves extract, have been incorporated in the inner infeed, increasing the oxidative stability of the encapsulated ALA-rich oil powders [8,10], but the incorporation of AA in ALA-rich oil microparticles, and in three different locations, has not been studied.

#### 3.5.3. AA Content

The AA recovery was greater than 80% in all the PWO microparticle systems (*p* > 0.05) with AA (PWO-C(AA)/SA, PWO-C/SA(AA) and PWO-C(AA)/SA(AA)) (Table 1). These results are in accordance with [33], where AA recovery values of 84% were reported when AA was encapsulated by spray drying with carboxymethyl cellulose.

#### 3.5.4. Moisture Content, Water Activity, Hygroscopicity

The moisture content of the PWO microparticles (Table 1) varied between 2.0 and 3.1% (*p* > 0.05), below the range of spray-drying powders used in the food industry (3–4% [27]). A low moisture content is an important factor in the shelf life of the spray-drying microparticles because it affects their handling and storage [26]. Water activity (aw) of the microparticles varied between 0.18 and 0.22, indicating, together with the low moisture content, a low risk of microbial deterioration. PWO-C microparticles showed hygroscopicity values of 23.4%, whereas all the microparticle systems with a SA outer layer showed values around 45%, indicating that hygroscopicity is polymer-specific.

#### 3.5.5. Morphology

Figure 3c–g shows the micrographs of the microparticle systems obtained by SEM. In general, both PWO-C (Figure 3c) and PWO-C/SA microparticles (Figure 3d) showed irregular shapes and sizes, with a relatively spherical appearance, with some shrinkage and tendency to agglomerate. Shrinkage can occur at the early stages of the drying process, at both high and low drying temperatures [34]. In this study, shrinkage occurred at low temperatures (114 °C) because water diffusion was slower and the microparticles have more time to shrink. Similar morphologies have been reported in oil microparticles with modified starch and SA as encapsulating agents [35,36]. Moreover, microparticle systems with AA addition, PWO-C(AA)/SA (Figure 3e), PWO-C/SA(AA) (Figure 3f) and PWO-C (AA)/SA(AA) (Figure 3g), showed spherical shape and smooth surface.

### 3.6. Thermal Analysis

The thermal profiles for C and the PWO microparticles systems (PWO-C, POW-C/SA, POW-C(AA)/SA, POW-C/SA(AA) and POW-C (AA)/SA(AA)) were determined (Figure S3). The melting points and Tg values are shown in Table 2. POW-C microparticles showed a sharp increase in the melting point when compared to C, due to the oil interaction with the hydrophobic moieties (octenyl succinate groups) of C. Two peaks, a small one at 166.74 °C, corresponding to C not interacting with oil, and a big one at 191.66 °C, corresponding to C interacting with the oil, were found in PWO-C microparticles.

In the case of microparticles with SA outer layer (POW-C/SA), there was a significant decrease in the melting point from 191.66 °C to 159.60 °C. This suggests that, even though microparticles with a well-defined SA outer layer were obtained by spray-drying, an intimate mixture of SA with C with a SA gradient concentration was formed. This result was supported by CLS microscopy (Figure 3a,b), where a representative microparticle (Figure 3b) is shown, with 0.68 µm of core diameter and 2.47 µm of total diameter. Considering that the mass ratio of (PWO-C) to SA used in the feed was about 1:1, the outer layer observed by CLSM must be C enriched with SA, with lower thermal stability. As only one fusion peak was found for this sample, this blend may behave as one phase, forming a SA concentration gradient with less SA towards the microparticle core. The addition of AA decreased even more the melting point of the microparticles to 139.13, 147.67 and 148.30 °C for PWO-C(AA)/SA, PWO-C/SA(AA) and PWO-C(AA)/SA(AA), respectively. The absence of the melting point of AA at 190 °C [37], and PWO-C at 191.66 °C, suggests that AA is also forming an intimate mixture within the microparticle components, either in the inner core or in the outer layer. This effect was more pronounced when the AA was localized in the inner infeed (139.13 °C, PWO-C(AA)/SA) compared with its localization in the SA outer layer (147.67 °C in PWO-C/SA(AA), and 148.3 °C in PWO-C(AA)/SA(AA). AA may disrupt the network where C surrounds the oil emulsion droplets, explaining the lowest melting point of the PWO-C(AA)/SA system and its destabilization. Furthermore, the higher the melting point the higher the phase transition enthalpy, thus explaining the stabilization of the formed microparticles.

As regards the Tg values, the PWO encapsulation decreased Tg of C from 60 °C to 53 °C. The incorporation of the SA outer layer (PWO-C/SA) raised the Tg up to 67 °C, as reported for polyvinyl acetate/sodium alginate mixtures by Caykara and Demirci [38]. The addition of AA decreased further the Tg in a similar rate when AA was added in the inner infeed (PWO-C(AA)/SA), in the outer infeed (PWO-C/SA(AA)) or in both (PWO-C(AA)/SA(AA)), showing Tg values of 50–52 °C.

### 3.7. Stability of Encapsulated PWO During Storage

The effect of AA on PWO oxidative stability was studied in PWO microparticles with a SA outer layer and AA (40 mM) incorporated in three different locations, the inner infeed (PWO-C(AA)/SA), the outer infeed (PWO-C/SA(AA)), and both infeed (PWO-C(AA)/SA(AA)), and compared with PWO microparticles without AA (PWO-C/SA, control). Figure 4b shows the time-course of formation of triacylglycerol dimers and polymers during storage of microparticles at 40 ± 1 °C (below Tg of the microparticles) in the dark. Determination of dimers and polymers have been largely proved to be an excellent measurement of lipid oxidation in oils rich in polyunsaturated fatty acids, as it is the case of WO, with around 15% of linolenic acid and 60% of linoleic acid. In fact, studies carried out in model unsaturated fatty acids and unsaturated oils have shown that the increase of polymerization compounds marks the end of the induction time and is concurrent with antioxidants exhaustion [39,40].

The specific location of AA influenced the oxidative stability of the encapsulated PWO, being the inner infeed the most effective location. These results were also consistent with the IP results already discussed, even though some differences can be expected due to the different temperature used. According to the increase of the content of dimers and polymers, the order of stability was PWO-C(AA)/SA >> PWO-C(AA)/SA(AA) > PWO-C/SA(AA) > PWO-C/SA. As suggested before, these results indicated that the greater protective effect of AA could be related to the removal of residual oxygen incorporated during the emulsification process [41]. A low protective effect was observed when AA was incorporated in the SA outer layer, attributable to limitation of oxygen diffusion through the microparticle matrix during storage [42].

## 4. Conclusions

A three-fluid nozzle was used to design PWO microparticles with a SA outer layer and an oxygen scavenger (ascorbic acid—AA) incorporated in three different locations of the microparticles (inner infeed, outer infeed and both infeed) by spray drying. The AA location in the PWO microparticles influenced the oxidative stability of the encapsulated PWO, and its location in the inner infeed greatly enhanced the oxidative stability of PWO, measured by the formation of secondary oxidation compounds (induction period and triacylglycerol dimers + polymers). AA removed the oxygen incorporated during the emulsion preparation due to high homogenization rates, which was the main factor involved in encapsulated PWO oxidation, whereas oxygen diffusion through the microparticle had minor relevance. However, the encapsulating agent features would determine the oxygen barrier properties of the microparticles. The specific-location of AA (inner infeed) may be a strategy to obtain stable spray-dried polyunsaturated vegetable oil-based microparticles until they are consumed and delivered at specific sites of the gastrointestinal tract intended by the outer polymer.

## Figures and Tables

**Table 1:** Characterization of PWO microparticle systems.

Parameter	PWO-C	PWO-C/SA	PWO-C(AA)/SA	PWO-C/SA(AA)	PWO-C(AA)/SA(AA)
EE (%)	80.2 ± 0.6 ^a^	94.0 ± 1.3 ^b^	93.8 ± 1.5 ^b^	93.9 ± 5.0 ^b^	92.9 ± 1.2 ^b^
IP (h)	0.99 ± 0.1 ^a^	1.84 ± 0.1 ^b^	4.56 ± 0.27^d^	1.17 ± 0.13 ^a^	2.6 ± 0.14^c^
Recovery AA (%)	-	-	86.3 ± 1.7 ^a^	85.2 ± 2.2 ^a^	83.8 ± 2.0 ^a^
Humidity (%)	2.3 ± 0.1 ^a^	2.0 ± 0.1 ^a^	2.50 ± 0.4 ^a^	2.3 ± 0.2 ^a^	3.1 ± 0.5 ^a^
a_w_	0.18 ± 0.0 ^a^	0.20 ± 0.01 ^a^	0.20 ± 0.01 ^a^	0.22 ± 0.02 ^a^	0.20 ± 0.02 ^a^
Hygroscopicity (%)	23.43 ± 1.3 ^a^	44.29 ± 1.6 ^b^	45.59 ± 1.5 ^b^	45.34 ± 1.3 ^b^	45.64 ± 1.6 ^b^

PWO: purified walnut oil; C: capsul; SA: sodium alginate; AA: ascorbic acid; EE: encapsulation efficiency; IP: induction period; a_w_: water activity; PWO-C: PWO microparticles with C; PWO-C/SA: PWO microparticles with C and a SA outer layer; PWO-C(AA)/SA: PWO microparticles with C, a SA outer layer and AA in the inner infeed; PWO-C/SA(AA): PWO microparticles with C, a SA outer layer and AA in the SA outer layer; PWO-C(AA)/SA(AA): PWO microparticles with C, a SA outer layer and AA in both infeed. Different letters (a–d) in the same row indicate significant (*p* < 0.05) differences between microparticle systems.

**Table 2:** Thermal analysis obtained in sealed samples by MDSC for C, SA and PWO microparticle systems.

Sample	Tg (°C)	M.P. (°C)	ΔH (J/g)
C	60.49	163.96	120.6
SA	n.d.	148.9	n.d.
PWO-C	53.23	166.74/191.66	4.48/n.d.
PWO-C/SA	67.13	159.60	94.29
PWO-C(AA)/SA	51.83	139.13	64.71
PWO-C/SA(AA)	51.67	147.67	75.29
PWO-C(AA)/SA(AA)	50.69	148.30	85.11

C: capsul; SA: sodium alginate; PWO purified walnut oil; AA: ascorbic acid; M.P.: melting point; ΔH: enthalpy for the melting transition; n.d.: non determined; PWO-C: PWO microparticles with C; PWO-C/SA: PWO microparticles with C and a SA outer layer; PWO-C(AA)/SA: PWO microparticles with C, a SA outer layer and AA in the inner infeed; PWO-C/SA(AA): PWO microparticles with C, a SA outer layer and AA in the SA outer layer; PWO-C(AA)/SA(AA): PWO microparticles with C, a SA outer layer and AA in both infeed.

## Supplementary Materials

The following are available online at https://www.mdpi.com/2076-3921/9/12/1272/s1, Figure S1: Location of AA in the PWO-C/SA microparticle system; Figure S2: Response surface graphics for droplet size (a–c) and TBARs (d–f) for PWO emulsion preparation; Figure S3: Thermal profiles for C and the PWO microparticles systems. a: Non-reversible heat flow. b: Reversible heat flow. C, black line; PWO-C microparticles, red line; POW-C/SA, blue line; POW-C(AA)/SA, green line; POW-C/SA(AA), purple line; POW-C (AA)/SA(AA), pink line; Table S1: Experimental design for PWO emulsion preparation. ANOVA for droplet size D_[4,3]_ and lipid oxidation (TBARs) of PWO emulsions; Table S2: Experimental design for PWO encapsulation by spray drying. ANOVA for encapsulation efficiency, induction period and yield of PWO-C microparticles.
