# Supplementary material for: Influence of the Location of Ascorbic Acid in Walnut Oil Spray-Dried Microparticles with Outer Layer on the Physical Characteristics and Oxidative Stability
Source: Antioxidants (Basel). 2020 Dec 14;9(12):1272. doi: 10.3390/antiox9121272 (PMC7765012; doi:10.3390/antiox9121272)
Supplement: Supplementary file 1 [file antioxidants-09-01272-s001.pdf]

Figure S1. Specific locations of ascorbic acid (AA) in the PWO-C/SA microparticle system

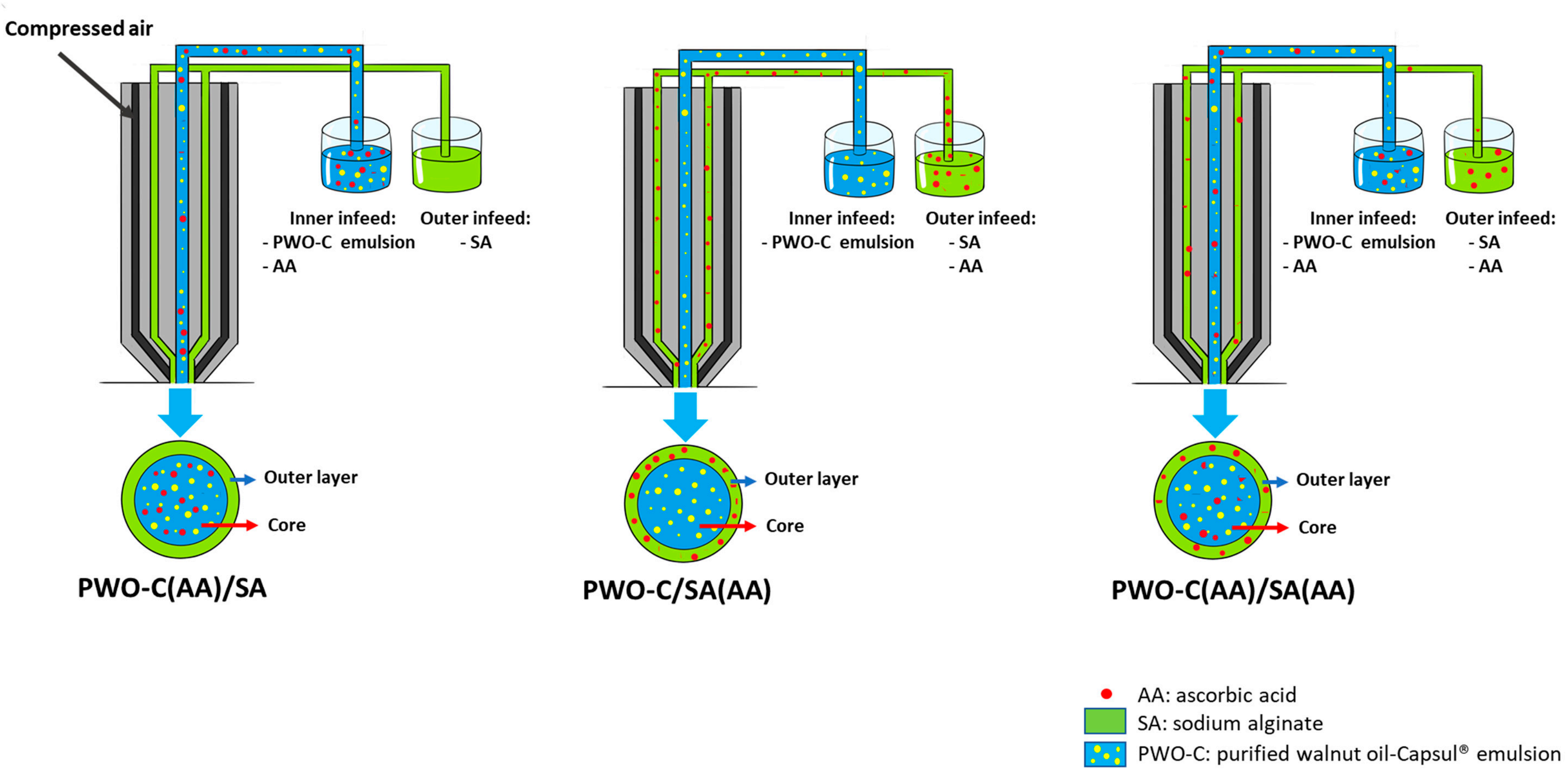

Figure S2. Response surface graphics for droplet size (a-c) and TBARs (d-f) for PWO emulsion preparation.

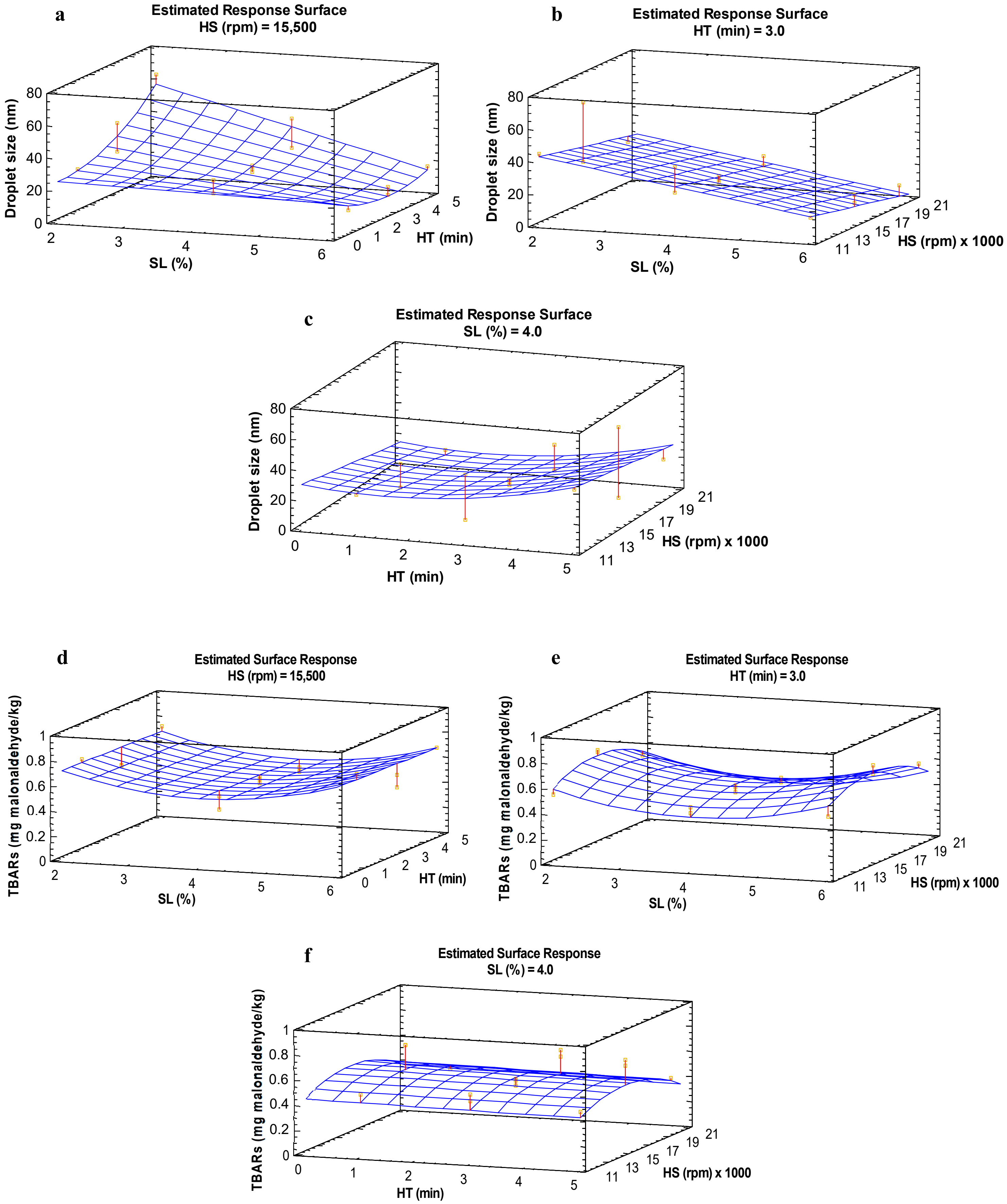

Figure S3. Thermal profiles for C and the PWO microparticles systems. a: Non-reversible heat flow. b: Reversible heat flow. C, black line; PWO-C microparticles, red line; POW-C/SA, blue line; POW-C(AA)/SA, green line; POW-C/SA(AA), purple line; POW-C (AA)/SA(AA), pink line.

a

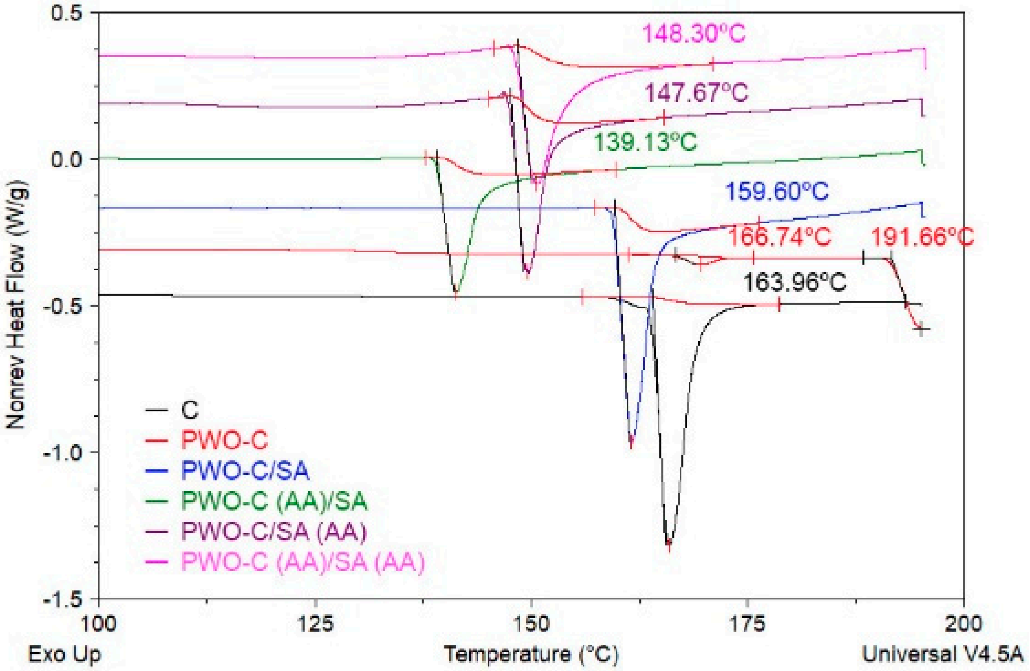

b

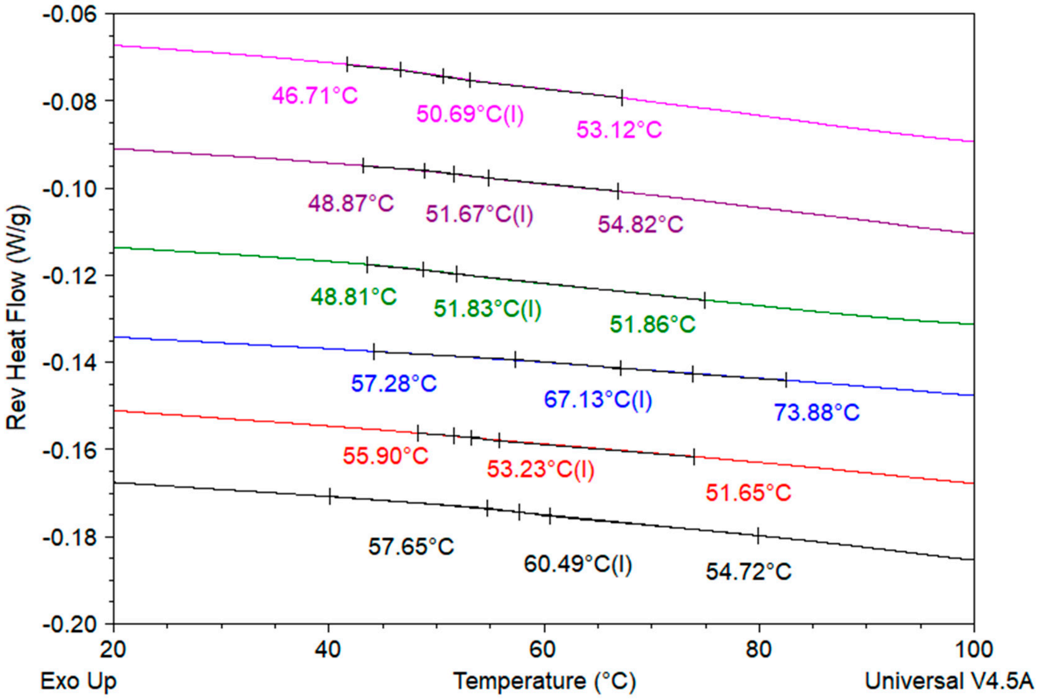

Table S1. Experimental design for PWO emulsion preparation. ANOVA for droplet size $D_{[4,3]}$ and lipid oxidation (TBARs) of PWO emulsions.

**Run**	**SL content (%)** (χ_1)	**HT (min)** (χ_2)	**HS (rpm)** (χ_3)	**Droplet size $D_{[4,3]}$**	**TBARs (mg MDA/kg oil)**
1	2.0	1	15500	27.3	0.743 ± 0.1
2	6.0	1	15500	12	0.745 ± 0.0
3	2.0	5	15500	63.7	0.729 ± 0.0
4	6.0	5	15500	17.2	0.684 ± 0.0
5	2.0	3	11000	44,7	0.556 ± 0.0
6	6.0	3	11000	15.6	0.503 ± 0.0
7	2.0	3	20000	27.3	0.549 ± 0.0
8	6.0	3	20000	10.5	0.605 ± 0.2
9	4.0	1	11000	25.8	0.508 ± 0.1
10	4.0	5	11000	41.5	0.464 ± 0.1
11	4.0	1	20000	17.6	0.401 ± 0.0
12	4.0	5	20000	23.6	0.432 ± 0.0
13	4.0	3	15500	19.6	0.525 ± 0.1
14	4.0	3	15500	21.6	0.511 ± 0.1
15	4.0	3	15500	23.8	0.473 ± 0.0

**ANOVA**	**Droplet size**		**TBARs**	
	Estimate	*p*-value	Estimate	*p*-value
βo	47.7781		-0.1667	
χ_1	-0.8812	0.0030*	-0.3175	0.6516
χ_2	3.825	0.0087*	-0.0055	0.3670
χ_3	-0.00135	0.0146*	0.000177	0.6216
χ_1^2	-	-	0.03937	0.0078*
χ_2^2	1.3219	0.0398*	-	-
χ_3^2	-	-	-5.75E-9	0.0141*
$\chi_1\chi_2$	-1.950	0.0177*	-	-
Lack of fit		0.1581		0.1736
R^2(adj. for d.f.)		89.70%		76.63%

SL: Soy lecithin HT: homogenization time; HS: homogenization speed; MDA: malondialdehyde; adj. for d.f.: adjusted for degrees of freedom. βo: intercept term; χ_1: SL content; χ_2: homogenization time; χ_3: homogenization speed; $\chi_1\chi_2$: interaction between SL content and homogenization time. Data are average ± standard deviation (n=3).

Table S2. Experimental design for PWO encapsulation by spray drying. ANOVA for encapsulation efficiency, induction period and yield of PWO-C microparticles.

Run	PWO:C ratio χ_1	Inlet air temperature (ºC) χ_2	EE (%)	IP (h)	Y (%)
1	1:1	120	72.4 ± 0.6	0.61 ± 0.0	19.8 ± 4.0
2	1:1	180	80.6 ± 0.3	0.7 ± 0.1	20.1 ± 0.0
3	1:5	120	81.0 ± 2.9	0.4 ± 0.1	36.2 ± 0.0
4	1:5	180	90.9 ± 1.1	0.49 ± 0.0	38.5 ± 1.9
5	1:3	113.7	83.2 ± 0.9	1.24 ± 0.1	25.4 ± 5.1
6	1:3	186.3	86.2 ± 1.4	0.22 ± 0.0	24.4 ± 3.3
7	1:0.58	150	66.6 ± 2.8	0.45 ± 0.0	16.1 ± 4.3
8	1:5.42	150	77.0 ± 5.7	0.57 ± 0.1	34.5 ± 0.0
9	1:3	150	76.4 ± 5.1	0.40 ± 0.0	33.3 ± 1.5
10	1:3	150	75.7 ± 0.2	0.42 ± 0.0	30.4 ± 3.6
11	1:3	150	77.9 ± 0.8	0.55 ± 0.1	30.1 ± 3.0
12	1:3	150	75.2 ± 2.5	0.47 ± 0.1	29.10± 0.6

**ANOVA**	**EE**		**IP**		**Y**	
	Estimate	*p*-value	Estimate	*p*-value	Estimate	*p*-value
βo	215.746		5.3933		14.794	
χ_1	5.5563	0.0022	0.0502	0.0289	4.1417	0.0012
χ_2	-2.1227	0.0066	-0.0579	0.0014	0.0063	0.8000
χ_1^2	-0.5485	0.0326				
χ_2^2	0.0074	0.0014	0.00016	0.0208		
Lack of fit		0.1208		0.1043		0.1685
R^2 (adj. for d.f.)		89.20%		79.96%		82.90%

PWO: purified walnut oil; C: capsul; EE: encapsulation efficiency; IP: induction period; Y: yield; adj. for d.f.: adjusted for degrees of freedom. βo: intercept term; χ_1: PWO:C ratio; χ_2: Inlet air temperature. Data are average ± standard deviation (n=3).
